# Early-Onset Myocardial Infarction in Adults Under 45: A Review of Five Cases

**DOI:** 10.7759/cureus.101119

**Published:** 2026-01-08

**Authors:** Samy Matta, Tambi Isaac, Roxana Lazarescu, Mujtaba Zafar, Hejmadi Prabhu

**Affiliations:** 1 Internal Medicine, Alexandria University, Alexandria, EGY; 2 Internal Medicine, Wyckoff Heights Medical Center, New York, USA; 3 Medical Academy, Kabardino-Balkarian State University, Nalchik, RUS; 4 Clinical Research, Xavier University School of Medicine, Elmont, USA; 5 Cardiology, Wyckoff Heights Medical Center, New York, USA

**Keywords:** left heart cath, multi-vessel coronary artery disease, myocardial infarction, patients younger than 40, premature cad

## Abstract

Myocardial infarction (MI) in individuals younger than 45 years is uncommon but carries significant clinical and psychosocial implications. Its presentation challenges traditional assumptions regarding cardiovascular risk and necessitates tailored approaches to diagnosis and management. We conducted a retrospective analysis of five patients aged <45 years admitted to a local hospital in New York with confirmed MI. Data included comorbidities, imaging, laboratory results, angiographic findings, and treatment modalities. Early-onset MI occurred predominantly in males, most of whom exhibited conventional risk factors. Coronary angiography revealed both single- and multi-vessel disease. Follow-up data were incomplete, with two patients lost to follow-up. These findings highlight the importance of recognizing conventional risk factors in younger populations and emphasize the need for individualized long-term management strategies.

## Introduction

Coronary artery disease (CAD) is a prevalent condition marked by impaired delivery of oxygen-rich blood to the myocardium, primarily due to atherosclerotic plaque formation along the coronary artery walls [1]. The progression of the plaque or the rupture of the plaque can lead to partial or complete obstruction of blood flow, ultimately resulting in myocardial ischemia, cellular death, and, in severe cases, infarction of cardiac tissue.

Although CAD is traditionally regarded as a disease of the elderly [2], it can also present in younger individuals as premature myocardial infarction (MI). This term applies when MI occurs before the age of 55 in men or 65 in women, representing a concerning deviation from the expected age-related pattern.

Younger patients with acute MI (AMI) often exhibit a distinct set of risk factors compared to older cohorts. Modifiable lifestyle behaviors such as tobacco use, recreational drug use (notably cocaine and marijuana), poor dietary habits, and medication non-adherence are prevalent. In some cases, underlying chronic conditions, such as systemic lupus erythematosus (SLE), seizure disorders, or undiagnosed hypercoagulable states, contribute to early-onset CAD [3,4]. There is limited characterization of angiographic findings in young patients; unlike the typical atherosclerotic burden seen in older adults, coronary angiography in young patients often reveals focal lesions with high thrombus burden and abrupt occlusions, rather than diffuse calcified disease [5,6].

These pathophysiological differences are reflected in their clinical presentations, which may include atypical symptoms, delays in seeking care, or substance-induced complications. Many patients present late in the disease course, often after using substances like opioids or benzodiazepines in attempts at symptom relief. This delay can result in extensive myocardial damage and increased periprocedural risk despite their relatively preserved ventricular function at baseline.

While percutaneous coronary intervention (PCI) often achieves excellent immediate results, the prognosis for young patients with AMI is far from benign. Studies show that many of these patients are at elevated risk for recurrent infarction, ventricular arrhythmias, and progressive heart failure if secondary prevention strategies are not rigorously implemented. Due to their young age, they require sustained, long-term follow-up, which can span decades. This results in a substantial cumulative financial burden on the healthcare system, particularly in settings where patients are underinsured or lack consistent access to care [7].

## Case presentation

We report a series of five cases involving patients aged ≤40 years with definitive MI, for whom past medical history, electrocardiogram data, and coronary angiography results were available.

Case 1

**Figure 1:**
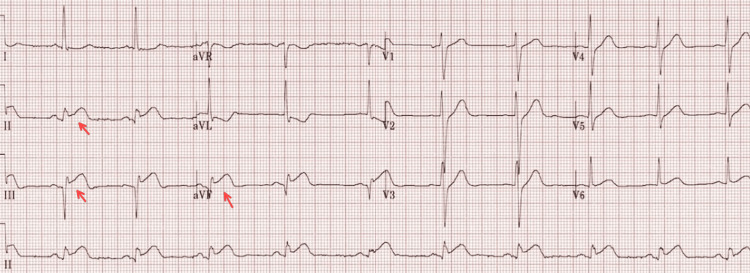
ST elevated in II, III, and aVF leads

This is a 40-year-old Hispanic male with a past medical history of hypertension, diabetes, and dyslipidemia, a nonsmoker with no known history of alcohol or recreational drug use, and a BMI of 34.1 who came to the ED with a chief complaint of vomiting and recurrent epigastric pain. In the ED, the patient experienced chest pain. An EKG was performed and showed ST elevation in the inferior leads (Figure 1, Table 1).

**Table 1:** Blood work LDL: low-density lipoprotein; HDL: high-density lipoprotein

Parameter	Patient Value	Reference Range
Troponin	7233 ng/L	3.0–58.9 ng/L
A1c	6.1%	4.0–5.6%
LDL	44 mg/dL	1–100 mg/dL
HDL	20 mg/dL	40–60 mg/dL

**Figure 2:**
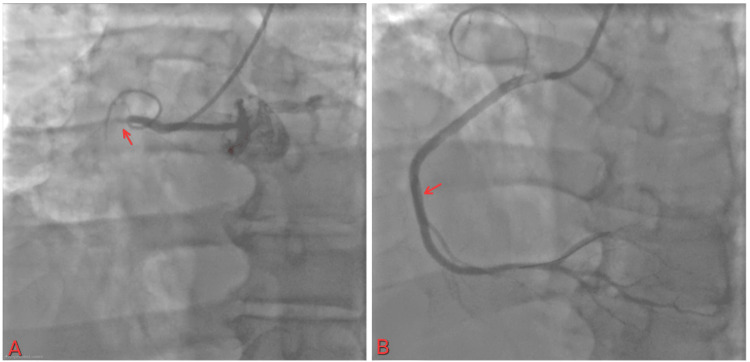
(A) 100% occlusion of proximal RCA with TIMI flow 0; (B) restoration of flow in RCA with TIMI flow III RCA: right coronary artery; TIMI: thrombolysis in myocardial infarction

The cardiac catheterization lab was activated. The patient was found to have a 100% occlusion of the proximal right coronary artery (RCA) (Figure 2), 80% in the mid-left anterior descending artery (LAD), and diffuse disease up to 80% in the distal circumflex. The lesion length was 60 mm, and a thrombus was seen. The patient received 38 and 26 mm stents in the proximal RCA, as it was the culprit lesion. He was later scheduled for staged PCI for other lesions.

He was transferred to the ICU. Follow-up troponin peaked a few hours after the intervention at 15536. ECHO showed inferior and inferio-lateral wall hypokinesis with EF 50-55%.

**Figure 3:**
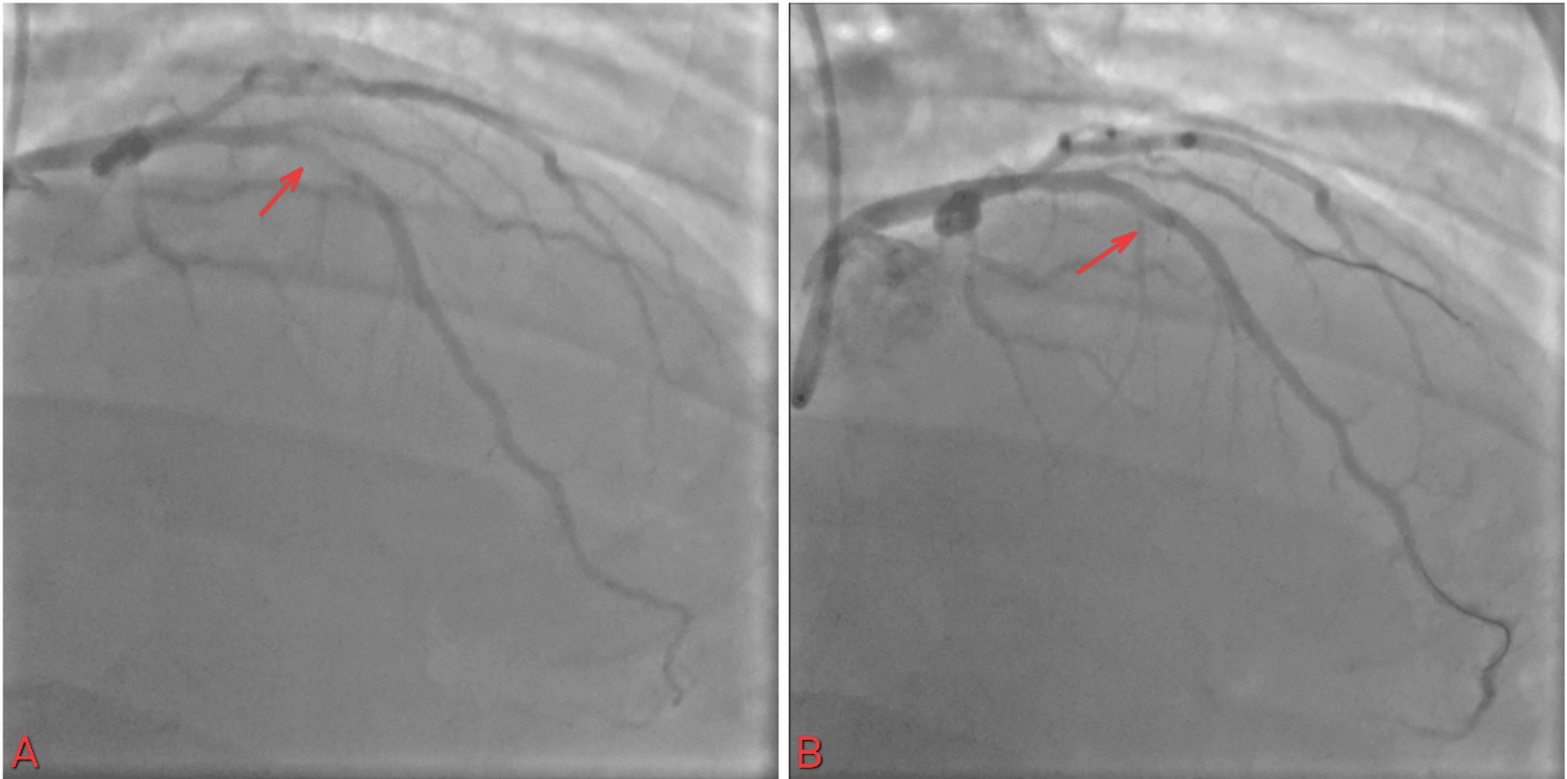
(A) Mid-LAD artery lesion; (B) stent placement and restoration of the flow LAD: left anterior descending artery

**Figure 4:**
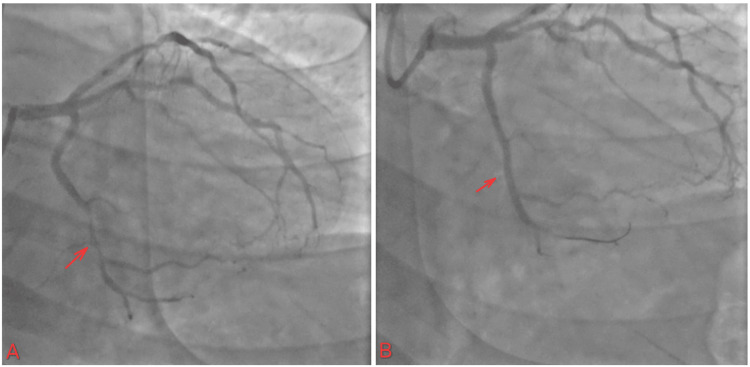
(A) Distal circumflex artery lesion; (B) restoration of the flow

The patient was discharged on day four. He came for a follow-up two weeks after discharge at the outpatient cardiology clinic. Nineteen days after the initial PCI, the patient underwent staged PCI with stent placement for both LAD (Figure 3) and Cx lesions (Figure 4). The patient continues to follow up as scheduled in the outpatient clinic.

Case 2

**Figure 5:**
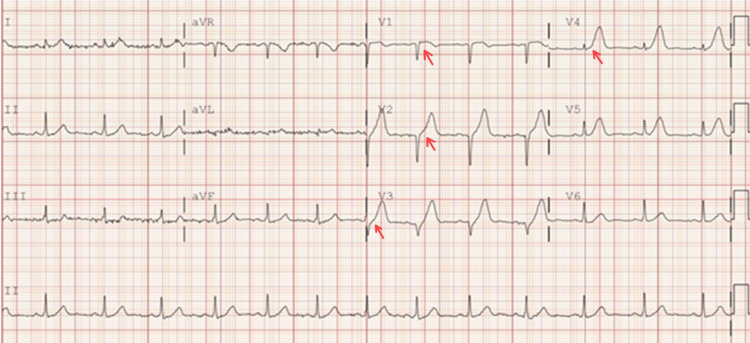
ST elevation V1-V4

This is a 37-year-old Polish male with dyslipidemia, a remote smoking history that is not clear, and a positive family history (father had MI in his early 50s) with a BMI of 26.4. This gentleman came to the emergency department with a complaint of central chest pain, with a severity of 10 out of 10. An EKG was performed and showed ST elevation in V1-V4 (Figure 5, Table 2).

**Table 2:** Blood work LDL: low-density lipoprotein; HDL: high-density lipoprotein

Parameter	Patient Value	Reference Range
Troponin	488 ng/L	3.0–58.9 ng/L
A1c	5.3%	4.0–5.6%
LDL	167 mg/dL	1–100 mg/dL
HDL	44 mg/dL	40–60 mg/dL

**Figure 6:**
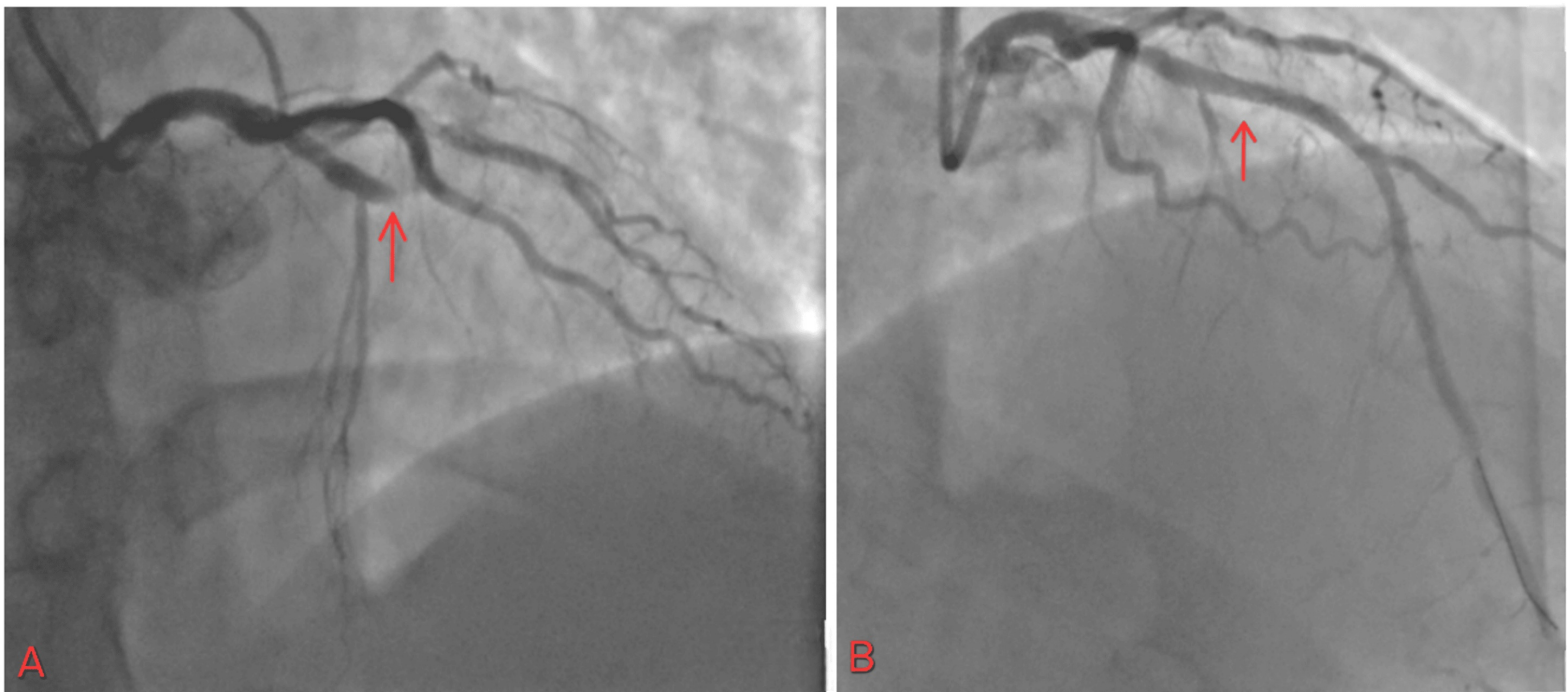
(A) 100% occlusion of mid-LAD with TIMI flow 0; (B) restoration of flow in LAD with TIMI flow III LAD: left anterior descending artery; TIMI: thrombolysis in myocardial infarction

The cardiac catheterization lab was activated. The patient was found to have 100% occlusion of the mid-LAD, moderate luminal irregularity up to the Cx, and mild luminal irregularity of less than 30%. Lesion length was 18 mm with thrombus present. Successful PCI with a drug-eluting stent to the mid-LAD (Figure 6).

The patient was transferred to the ICU. Follow-up troponin was trending up and peaked six hours after the intervention at more than 125,000. The ICU course was significant for hypotension. ECHO showed mid-anterior, anterior-septal, and inferior wall hypokinesia and apical anterior, lateral, inferior, and septal wall hypokinesis with EF 30-35%. Due to low EF, the patient was discharged with a life vest after four days. The patient came for follow-up in the Heart Failure Clinic twice after discharge and came to the Cardiac-Electrophysiology Clinic for life vest interrogation. The patient has not returned for follow-up appointments since one month after discharge. Efforts to reach the patient for continued care have been unsuccessful.

Case 3

**Figure 7:**
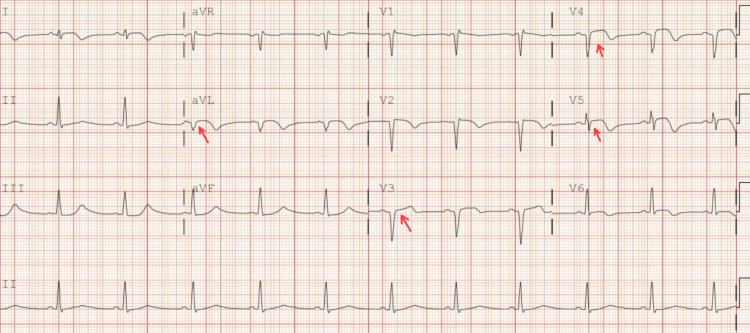
EKG showed ST-segment elevation in aVL, V3-V4-V5

This is a 40-year-old Hispanic male with a past medical history of hypertension, a nonsmoker with no history of recreational drug use, and a BMI of 25.4 who came to the emergency department with a complaint of crushing chest pain with a severity of 10 out of 10. The EKG showed ST-segment elevation in the anterolateral leads (Figure 7, Table 3).

**Table 3:** Blood work LDL: low-density lipoprotein; HDL: high-density lipoprotein

Parameter	Patient Value	Reference Range
Troponin	32 ng/L	3.0–58.9 ng/L
A1c	4.9%	4.0–5.6%
LDL	90 mg/dL	1–100 mg/dL
HDL	47 mg/dL	40–60 mg/dL

The cardiac catheterization lab was activated. The patient was found to have 100% occlusion of the proximal LAD, 60% of proximal Diagonal 1, and a large RCA with moderate diffuse disease of the right posterolateral artery. A successful PCI with a 38 mm stent was placed in the proximal LAD (the culprit vessel). The patient was subsequently transferred to the ICU. Follow-up troponin was trending up and peaked at 45648. The echocardiogram showed anterior and anteroseptal wall hypokinesis. The apex was hypokinetic with an ejection fraction of 45-50%. The patient was discharged on the third day for follow-up at the outpatient clinic.

A follow-up echocardiogram three months after the MI showed an ejection fraction of 35%-40%. Another echocardiogram performed six months after the MI also showed an ejection fraction of 35-40%. The most recent one was performed 18 months after the MI and showed a normal ejection fraction with no regional wall motion abnormality.

Case 4

**Figure 8:**
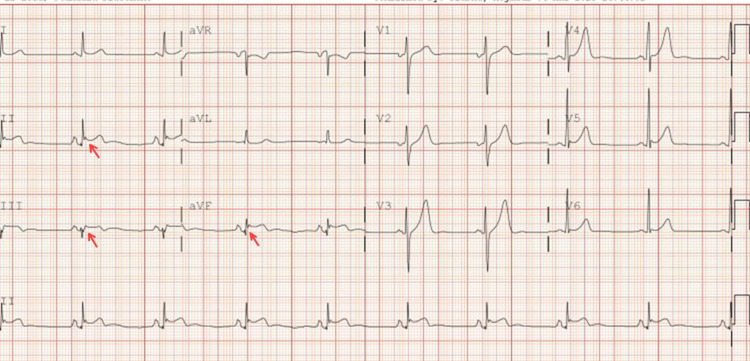
EKG showed ST elevation in inferior leads II, III, and aVF

Our patient is a 34-year-old African American male with a history of active smoking (one pack every five to seven days since the age of 20 and marijuana one to two cigarettes a day), BMI: 29.4, otherwise nonsignificant, who presented initially to the emergency department with a complaint of unprovoked left-sided chest pain radiating to the left arm. In the ED, blood pressure was 167/97. The EKG showed ST elevation in the inferior leads (Figure 8, Table 4).

**Table 4:** Blood work LDL: low-density lipoprotein; HDL: high-density lipoprotein

Parameter	Patient Value	Reference Range
Troponin	1202 ng/L	3.0–58.9 ng/L
A1c	6.1%	4.0–5.6%
LDL	166 mg/dL	1–100 mg/dL
HDL	44 mg/dL	40–60 mg/dL

**Figure 9:**
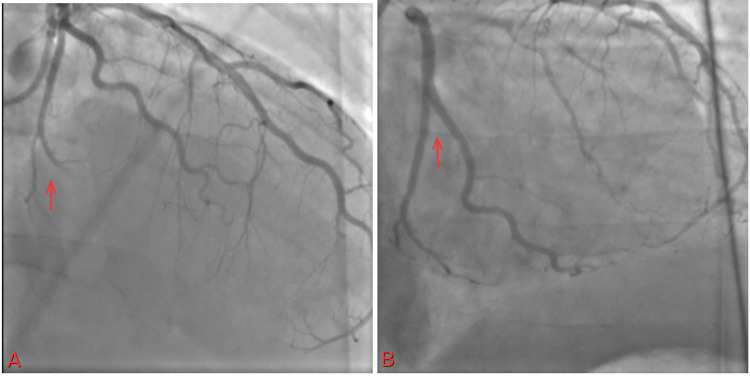
(A) 100% occlusion of OM3 and distal branch OM2; (B) improvement of flow after use of a 1.5 balloon OM3: obtuse marginal artery 3; OM2: obtuse marginal artery 2

The cardiac catheterization lab was activated. The patient was found to have 100% thromboembolic occlusion of the obtuse marginal artery 3 (OM3) and the obtuse marginal artery 2 (OM2) (Figure 9).

A plain balloon intervention for OM3 was performed using a 1.5 balloon, which improved flow in the vessel. No stent was placed, as the vessel was less than 2 mm in caliber. Follow-up troponin was trending up and peaked at 44176.

An echocardiogram was performed subsequently and showed no wall motion abnormality with an EF of 55-60%. Due to concern about the thromboembolic cause of MI, contrast ECHO was performed and showed no evidence of noncompaction. Hematology was consulted, and a comprehensive hypercoagulable workup was performed, yielding negative results.

Due to concern about paradoxical embolization, a saline contrast echocardiogram was performed with no right-to-left shunt. The patient was discharged on the third day with a follow-up appointment within two weeks after discharge at the cardiology clinic; however, the patient never showed up, and attempts to contact the patient were unsuccessful.

Case 5

**Figure 10:**
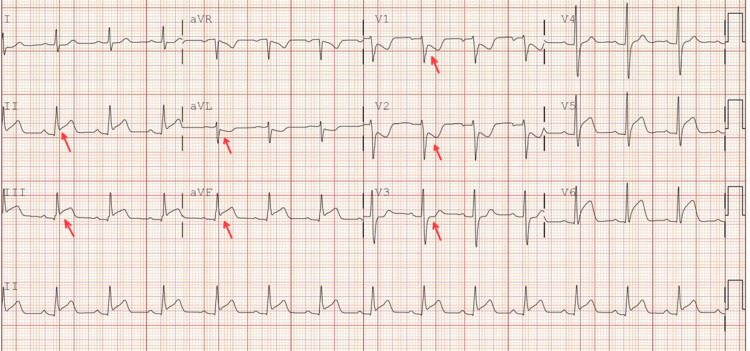
Inferior-posterior ST elevations with reciprocal ST depression in V1-V3

A 40-year-old Hispanic male with a past medical history of hypertension, asthma, active smoking (two packs per day since the age of 16), seizure disorder (non-compliant with medications), and a history of lumbar fusion surgery two days prior, BMI: 28.7, presented to the hospital with a complaint of chest pain. This prompted an EKG to be performed, which showed inferior-lateral ST elevations with reciprocal ST depression in V1-V3 (Figure 10).

Given the recent spinal surgery, the multidisciplinary team, including cardiology, neurosurgery, and the primary medical team, and the patient's family engaged in a comprehensive discussion to determine the safest course of action, balancing urgent reperfusion therapy against potential bleeding risks related to the recent spinal procedure. The team weighed options such as dual antiplatelet therapy (DAPT), PCI, and potential surgical adjustments (Table 5).

**Table 5:** Blood work LDL: low-density lipoprotein; HDL: high-density lipoprotein

Parameter	Patient Value	Reference Range
Troponin	280 to 48,232 ng/L (1 hour later)	3.0–58.9 ng/L
A1c	5.7%	4.0–5.6%
LDL	147 mg/dL	1–100 mg/dL
HDL	38 mg/dL	40–60 mg/dL

**Figure 11:**
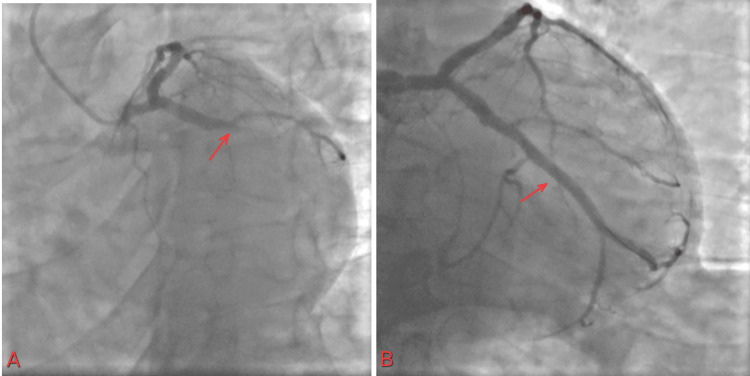
(A) 100% occlusion of prox Cx with TIMI flow 0; (B) restoration of flow in proximal Cx with TIMI flow III TIMI: thrombolysis in myocardial infarction

Given the STEMI state and rising troponin, the patient agreed to go for PCI, which showed elevated left ventricular end diastolic pressure (23 mmHg). The prox circumflex showed 100% stenosis, prox RCA showed 85% stenosis, mid-RCA showed 85% stenosis, distal RCA showed 70% stenosis, and LAD showed mild luminal irregularity, less than 30% (Figure 11).

**Figure 12:**
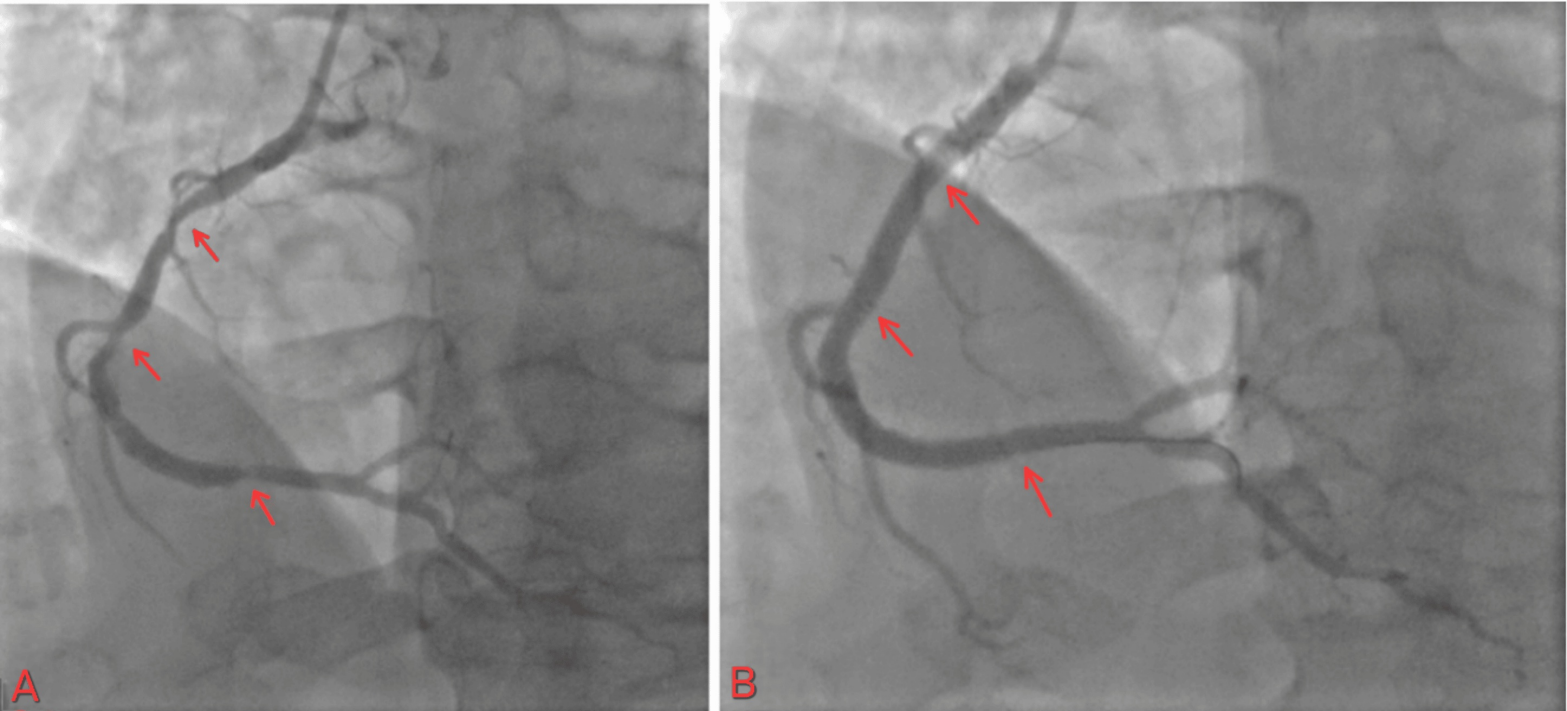
(A) Proximal and mid-RCA 85% stenosis and 70% stenosis in the distal RCA; (B) the flow after multiple stent placements in the RCA to cover the lesions

The patient started on DAPT with close monitoring of neurological status. An echocardiogram was performed with an EF of 50%-55%. The patient was discharged on the sixth day, and a staged PCI was scheduled one month later for RCA PCI. The patient returned for outpatient follow-up and underwent staged PCI for RCA disease. During the procedure, multiple stents were placed to ensure adequate coverage of the affected segment, optimizing coronary perfusion. The patient continues to follow up as scheduled in the outpatient clinic (Figure 12).

## Discussion

While traditionally considered a disease of older adults, CAD increasingly affects younger individuals, including patients under 45 years of age [8]. The cases presented here demonstrate that significant MI can occur in young patients with varying risk profiles. Early-onset MI in young adults presents a significant clinical challenge. Timely diagnosis is often hindered by the prevailing misconception that MI rarely affects this age group, resulting in delays in recognition and management. Furthermore, the presence of comorbidities in these patients compounds the complexity of care and may limit their ability to fully contribute to society during their most productive years.

Risk factors in young CAD patients

Despite their young age, many of these patients had traditional cardiovascular risk factors such as dyslipidemia, hypertension, smoking, diabetes, and a family history of premature CAD. These findings are consistent with prior studies showing that traditional risk factors still play a substantial role in early-onset CAD [9,10]. However, some patients in this series had limited or unclear risk factors, suggesting potential contributions from genetic predisposition, environmental exposures, or yet unidentified factors. A similar case series presented by Tudurachi et al. [11]discussed different risk factors that contributed partially or fully to MI, including premature atherosclerosis, substance use, human immunodeficiency virus (HIV)-related coronary disease, and spontaneous coronary artery dissection.

Challenges in risk prediction

Standard cardiovascular risk calculators, such as the Framingham Risk Score and the Atherosclerotic Cardiovascular Disease (ASCVD) calculator, may underestimate risk in younger individuals due to their age-dependent weighting [12]. For instance, the Framingham Risk Score calculates the risk for patients aged 30-79, but it has limited comorbidity inclusion; on the other hand, the ASCVD calculator only provides 10-year risk estimates for the US cohort between 40 and 75 years of age. QRISK3 is another risk calculator that allows an earlier age range (typically 25-84) and incorporates more comorbidities and risk factors, but it is tailored only to the UK population. This calls for the development or refinement of risk assessment tools that incorporate genetic markers, family history, and novel biomarkers to better identify high-risk young adults before a major cardiovascular event occurs. Emerging data on subclinical atherosclerosis detection using coronary artery calcium (CAC) scoring or vascular imaging may offer additional predictive value in select patients [13].

Loss of follow-up and its clinical implications

A notable finding in this series is the significant rate of loss to follow-up, with two of the five patients failing to return for scheduled outpatient visits. This raises important concerns about the psychological, socioeconomic, and systemic factors affecting adherence to long-term care in young MI survivors. Younger patients may experience denial, depression, anxiety, or financial barriers that interfere with ongoing care. Early psychiatric evaluation, patient education, and social work support should be integrated into the care plan to address these challenges and improve long-term outcomes.

Psychosocial barriers such as unemployment, poor health literacy, or untreated mental health conditions frequently contribute to poor adherence to medications, follow-up visits, and lifestyle modifications. Young patients are significantly less likely than older adults to attend cardiac rehabilitation or adhere to guideline-directed therapy, despite being the ones who would benefit the most from long-term preventive care [14].

There is thus a critical need for multidisciplinary, patient-centered strategies aimed at improving secondary prevention in this population. These must address not only medical risk factors but also behavioral, psychological, and socioeconomic determinants of health. Integration of addiction counseling, mental health services, and tailored education on medication adherence should be standard components of post-AMI care for young adults.

Potential for long-term complications

The spectrum of myocardial injury ranged from preserved ejection fraction to significant heart failure with reduced ejection fraction. As demonstrated in these cases, some patients with initially preserved cardiac function experienced progressive ventricular dysfunction on serial echocardiograms, underscoring the need for careful long-term surveillance even in apparently stable patients.

Proposed preventive strategies

To address this emerging public health concern, early screening and aggressive management of modifiable risk factors are critical. This includes routine lipid screening, smoking cessation counseling, lifestyle modifications (such as structured exercise programs), and strict blood pressure and glycemic control. The role of early initiation of lipid-lowering therapy in young adults with borderline risk remains an area for further research [15]. Additionally, educational efforts targeting primary care providers may help facilitate earlier identification of high-risk individuals.

Future directions

Further research is needed to better understand the genetic, molecular, and environmental contributors to premature CAD. Large-scale registries and prospective studies focusing on young MI patients may provide insight into optimal preventive and therapeutic strategies. Multidisciplinary care teams, including cardiology, primary care, psychiatry, and social work, are essential to address the complex needs of this unique patient population.

## Conclusions

This case series highlights five patients under the age of 45 who experienced MI as the initial manifestation of CAD, with presentations both in the presence and absence of conventional risk factors. While further evaluation for alternative pathophysiological mechanisms, such as vasculitides, infections, and hypercoagulable states, was not comprehensively pursued, the findings underscore the complexity of premature MI. By analyzing clinical presentations, procedural outcomes, and long-term risk profiles, we contribute to the growing body of evidence that emphasizes the need for targeted, longitudinal strategies to reduce morbidity and mortality in this population. Multidisciplinary approaches are essential to minimize loss to follow-up, enhance compliance, and provide education regarding the nature of this condition, with a focus on modifying preventable risks while addressing non-modifiable factors.

Premature MI in patients under 45 is an increasingly recognized clinical challenge with substantial implications for long-term health and societal productivity. Although traditional risk factors remain central, additional contributors are likely involved. Early recognition, aggressive risk factor modification, psychological support, and coordinated multidisciplinary care are critical to improving outcomes and reducing the burden of disease in this vulnerable group.
